# Applying sigma metrics to assess quality control processes in the transfusion transmissible infection screening laboratory of a blood center

**DOI:** 10.1371/journal.pone.0312422

**Published:** 2024-10-24

**Authors:** Sonu Bhatnagar, Sten Westgard, Nguyen Thi Thanh Dung, Tran Ngoc Que, Bach Quoc Khanh, Nguyen Ha Thanh

**Affiliations:** 1 Transfusion Medicine, Abbott Laboratories, Singapore, Singapore; 2 Westgard QC, Inc., Madison, WI, United States of America; 3 National Institute of Hematology and Blood Transfusion, National Blood Center, Ha Noi, Vietnam; Centers for Disease Control and Prevention, UNITED STATES OF AMERICA

## Abstract

In the field of healthcare, quality and efficiency are of paramount importance to ensure the provision of safe and reliable diagnostic services. Blood screening laboratories play a critical role in detecting and preventing the spread of infectious diseases, ensuring the safety of blood transfusions, and supporting medical diagnoses. To enhance the quality of processes in such laboratories, the Six Sigma methodology has gained significant recognition for its ability to systematically identify and minimize variations, thereby improving overall efficiency and reducing errors. This retrospective study aims to explore the application of Six Sigma metrics in the context of blood screening laboratories, providing an in-depth analysis of its implementation, benefits, and challenges. The performance of three serology assays, i.e., anti-HCV, HIV Ag/Ab combo, and HBsAg, using internal quality control (IQC) daily data extracted from six Alinity i instruments (Abbott GmbH, Germany), from February to April 2023, was evaluated. Mean, standard deviation (SD), and coefficient of variation (CV%) was calculated for positive controls. Bias was calculated using peer group data. Sigma metrics were calculated using allowable total error (TEa %) based on difference between the observed mean of the positive control and the s/co cut-off of assay. The observed CV% for positive controls was ≤10%. The TEa% ranged from 66% to 79% for the analytes using the observed mean. All the assays showed Six Sigma performance (σ>6) with and without bias. The study observed that the serology assays showed very high sigma values (σ>6) and thus, simplified statistical quality control (SQC) design based on Westgard Sigma rules could be implemented without compromising blood safety.

## Introduction

Infectious diseases pose significant threats to public health, particularly when transmitted through blood transfusions [1]. Accurate serology assays for detecting pathogens like Hepatitis C Virus (HCV), Hepatitis B Virus (HBV), and Human Immunodeficiency Virus (HIV) are critical in ensuring the safety of blood products [2]. However, maintaining the quality control of these assays presents challenges due to factors such as variability in reagents, equipment, and operator proficiency.

According to the World Health Organization (WHO), approximately 1.3 million people were newly infected with HIV in 2022 [3], and an estimated 1.2 million new HBV infections [4] occur annually globally. Furthermore, HCV affects an estimated 50 million people worldwide [5], leading to significant morbidity and mortality if left untreated. Despite the advent of new technologies such as nucleic acid testing (NAT), and pathogen inactivation/reduction (PRT), accurate and reliable serology testing is still essential for early detection and timely intervention to prevent disease transmission through blood transfusions in most of the countries [6].

The Six Sigma methodology offers a systematic approach to address these challenges by providing a framework for process improvement and quality management [7,8]. Building upon successful applications in other clinical laboratory tests, such as clinical chemistry [9] and hematology [10] assays, Six Sigma analysis has demonstrated its effectiveness in enhancing the quality control of diagnostic procedures [11]. For instance, a study by Ibrahim et al. (2022) [12] applied lean six sigma principles to evaluate the performance of hemoglobin assays in a clinical laboratory setting, achieving significant improvements in turnaround time and result reliability. Similarly, Panda et al. (2023) [13] utilized Six Sigma analysis to optimize the quality control processes of chemistry assays, resulting in reduced error rates and enhanced laboratory efficiency. This research paper aims to investigate the application of Six Sigma analysis in assessing the quality control of infectious diseases serology assays in blood screening laboratories. Through a comprehensive evaluation of key metrics such as quality control imprecision and bias, error rates, and sigma levels, this study seeks to provide insights into the effectiveness of current quality control measures and opportunities for optimization. The objectives of this research include—evaluating the performance of serology assays for HCV, HBV, and HIV using Six Sigma metrics, and proposing strategies for optimizing statistical quality control (SQC) and thereby, reducing the risk of false-negative or false-positive results in blood screening. By addressing these objectives, this study aims to contribute to the advancement of quality assurance practices in blood screening laboratories and ultimately enhance the safety of blood transfusions. The findings of this research have the potential to impact decision-making processes, guide resource allocation, and improve patient outcomes in the field of infectious diseases serology testing.

## Materials and methods

### Ethical considerations

Ethical approval was obtained from the National Institute Hematology & Blood Transfusion (NIHBT) Ethics Committee. The results of quality control (QC) samples were utilized and therefore no patient consent was required.

### Study setting

This retrospective study was conducted in a Transfusion Medicine Laboratory at NIHBT, a large academic tertiary hospital in Vietnam, accredited to ISO 15189. The laboratory uses six identical Alinity i immunoassay analyser (Abbott GmbH, Germany) labeled Analysers 1 to 6, running in parallel. Some tests are performed on all the analysers. The laboratory operates in two shifts (morning and evening), with three analysers used per shift. Internal Quality Control (IQC) with kit control and one in-house control is performed during every shift, and Westgard classic rules are applied to accept or reject the run.

### Study design

A retrospective analysis was conducted on three analytes: antibody to hepatitis C virus (anti-HCV), antibodies to human immunodeficiency virus (HIV) type 1 and/or type 2 and HIV p24 antigen (HIV Ag/Ab combo), and hepatitis B surface antigen (HBsAg). IQC data from the Alinity i immunoassay analyser IT middleware system (Abbott GmbH, Germany) was used, covering February to April 2023. The Alinity i employs chemiluminescence microparticle immunoassays (CMIA) for detecting antigens or antibodies in human serum and plasma. All three assays use the same CMIA principle and were calibrated according to the manufacturer’s instructions.

The laboratory used Abbott QC materials (kit controls) and an in-house control (positive sample) to perform IQC testing. Two levels of kit controls, one negative and one positive, were performed twice daily for each analyte, except for HIV, which had one negative and three positive controls. All controls were operated according to the manufacturer’s instructions.

### Statistics

Imprecision was assessed from the positive Kit controls run daily for each month. Three-month imprecision values were used to calculate average imprecision. The CV% was determined using the following formula:

CV%=100×(standarddeviation/mean).

Since there is no universally accepted gold standard or true reference method, it is difficult to obtain an accurate bias estimate. Thus, Sigma was calculated simply as the ratio of TEa% to CV% [14]. However, bias was computed from the External Quality Assurance (EQA) report [15] of the peer group data, using the following formula: Bias (%) = [(mean of all laboratories using same instrument and method- lab mean)/mean of all laboratories using same instrument and method]×100. This bias was then used for comparison between Sigma values with and without bias.

The expected mean (${\bar{x}}_{\exp}$) of the positive control was calculated using the control range (minimum and maximum) provided by manufacturer [16]. The observed mean (${\bar{x}}_{\mathrm{obs}}$) was calculated as the average of three-month mean. The distance between the means of the positive control (${\bar{x}}_{\exp}$ and ${\bar{x}}_{\mathrm{obs}}$) and the 1.0 cutoff was taken as TEa (TEa_exp_ and TEa_obs,_ respectively). This is based on the understanding that results with low positive S/CO should not be missed and reported as negative (i.e., a false negative) [14].

Sigma metrics calculation

The sigma value was calculated using the standard equation:

Sigma=TEa%‐Bias%/CV%(withandwithoutBias%).

The performance evaluation standards were divided into six levels: world-class: σ≥6; excellent: 5≤σ<6; good: 4≤σ<5; marginal: 3≤σ<4; poor: 2≤σ<3; Unacceptable: σ<2. A sigma value of 3σ was considered the minimum acceptable limit. Quality control rules were selected based on Westgard Sigma multi-rules [11] using observed sigma values to calculate the number of out-of-control events retrospectively.

Data capture and statistical analyses were performed using Windows® 10 and Microsoft Excel (Microsoft Corporation, Washington, United States). Microsoft Excel was used to calculate mean, standard deviation, and analytical sigma-metrics.

## Results

The Sigma metrics for the three analytes, calculated from internal QC data (February-April 2023), are shown in Table 1. HBsAg and anti-HCV were run on analysers 1–4, while HIV Ag/Ab was run on analysers 1,3,5, and 6. The sigma value was >6 for all the analytes using TEa%/CV%. Bias% using the EQA report was -6.9 for anti-HCV, -4.8 for HBsAg, and -8.3 for HIV Ag/Ab. The sigma value was >6 for all the analytes when Bias% was included in the calculation (data not shown). Our results indicate that all the three analytes showed imprecision ≤ 10% which is within the manufacturer’s recommended limits (≤10%CV for any positive controls) [16]. The TEa% calculated using the observed mean (TEa_obs_ %) ranged from 66% to 79% for the controls analysed. The TEa% calculated using the expected mean (TEa_exp_ %) from the manufacturer- provided range was close to TEa_obs_ % for HBsAg and anti-HCV, but not for HIV Ag/Ab (S1 Table). During the study period, five reagents lots were used for HBsAg, and three for anti-HCV and HIV Ag/Ab respectively.

**Table 1:** Mean, CV%, TEa_obs_% and sigma for 3 Serology analytes (Feb-Apr 2023).

*Analyte	^IQC	#Analyser	N	${\bar{x}}_{\mathrm{obs}}$	CV%	TEa_obs_ %	Sigma	~NOPSx
**HBsAg**	**POS**	1	98	3.63	5.17	72.5	14.0	7.1
		2	82	3.56	3.09	71.9	23.3	4.3
		3	86	3.49	3.11	71.4	22.9	4.4
		4	57	3.57	4.20	72.0	17.1	5.8
**Anti-HCV**	**POS**	1	83	4.78	6.43	79.1	12.3	8.1
		2	69	4.83	7.38	79.3	10.7	9.3
		3	71	4.72	5.78	78.8	13.6	7.3
		4	55	4.65	5.40	78.5	14.5	6.9
**HIV Ag/Ab**	**POS3**	1	90	2.93	3.11	65.8	21.1	4.7
		3	91	3.01	2.98	66.8	22.4	4.5
		5	58	3.06	2.32	67.4	29.0	3.5
		6	75	2.99	2.61	66.5	25.5	3.9
**HIV Ag/Ab**	**POS2**	1	90	3.23	6.04	69.1	11.4	8.7
		3	91	3.35	4.09	70.1	17.2	5.8
		5	58	3.39	3.90	70.5	18.1	5.5
		6	75	3.40	3.76	70.6	18.8	5.3
**HIV Ag/Ab**	**POS1**	1	90	4.61	5.96	78.3	13.1	7.6
		3	91	4.67	3.90	78.6	20.2	5.0
		5	58	4.77	4.74	79.0	16.7	6.0
		6	75	4.72	4.53	78.8	17.4	5.7

*Abbott Alinity i HBsAg, Anti-HCV, HIV Ag/Ab Combo, Reagent Kits; ^Alinity i HBsAg, Anti-HCV, HIV Ag/Ab Combo Positive Kit controls; # Alinity i analyser 1–6; `Observed mean; ~Normalized Operating Specifications x-co-ordinate.

**Fig 1:**
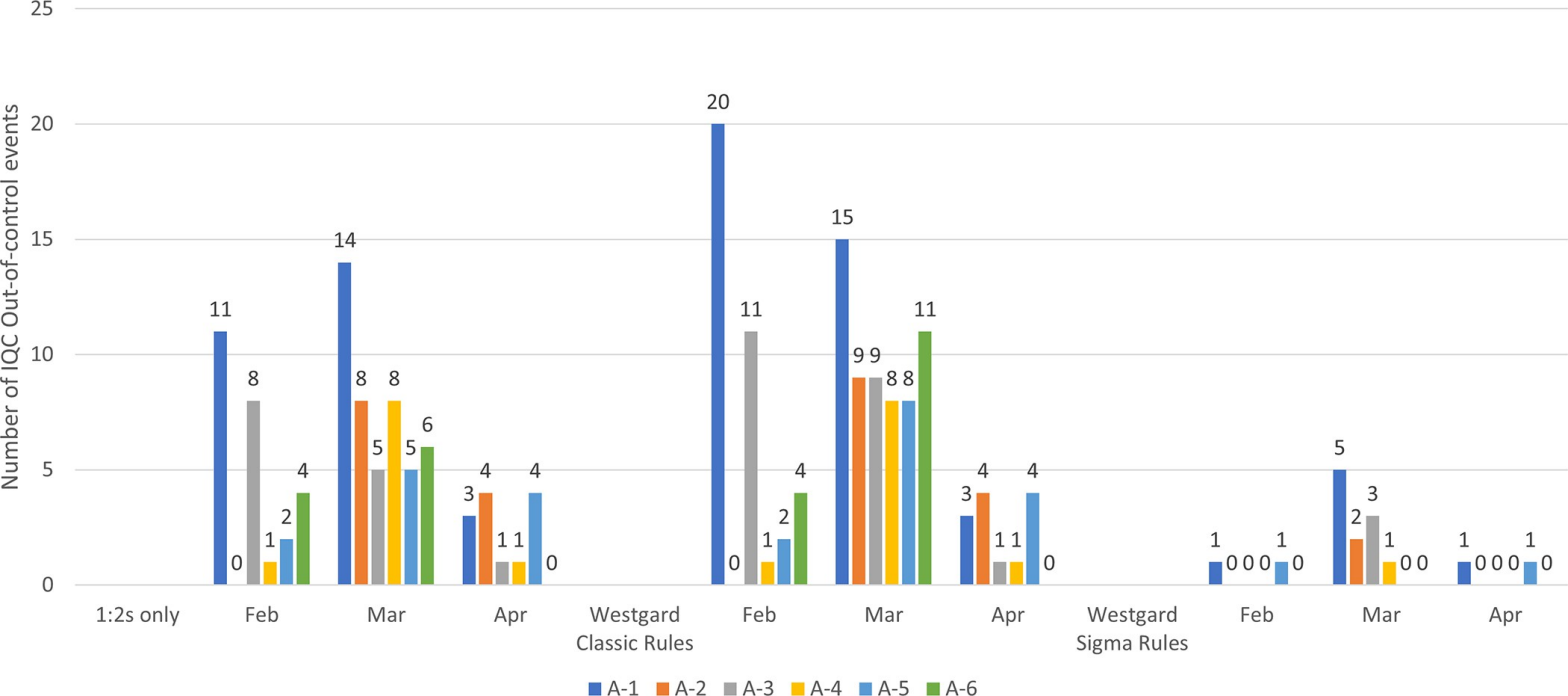
Total number of IQC “out-of-control events” on Analyzer 1–6 for 3 Serology analytes. A1-A6 indicates Analyzer 1 to 6; Each bar represents the total number of out-of-control events for all three analytes on a particular analyzer in the specified month, based on three different QC rules: 1:2s only, Westgard classic rules, and Westgard sigma rules.

The total number of IQC out-of-control events for all the analytes on each analyser is shown in Fig 1. There was a 87% reduction in out-of-control events during the three-month study period when switching from Westgard classic rules to Westgard sigma rules (S2 Table). In April, there was one “out-of-control” IQC result for HBsAg on analyer 2 due to incorrect QC loading, which was excluded from the sigma calculations (S1 Table). The CV% for HBsAg on analyser 1 was higher in February (8.55%) compared to March (3.46%) and April (3.5%). There was a random error (1:3s) due to sample aspiration on analyser 1 in February which accounted for higher CV%. The CV% for anti-HCV on analyser 2 was 13.24% in February due to reagent lot change. Overall, the CV% for anti-HCV was slightly higher in February and March compared to April across all the analysers (1–4)**.** For HIV Ag/Ab, Pos 1 showed 8.82% imprecision on analyser 1 in February and 7.23% on analyser 5 in March. Pos 2 on analyser 1 in February had 8.83% imprecision due to the use of two different reagent lots (S1 Table).

## Discussion

The safety and efficacy of blood products is of paramount importance in transfusion services. Infectious serology screening assays play a pivotal role in detecting infectious agents and ensuring the quality of blood donations [6]. To assess the performance and accuracy of these assays, the application of sigma metrics analysis could be a potential powerful tool [7]. Sigma metrics provide a comprehensive and standardized approach to evaluate the analytical quality of laboratory tests, thereby aiding in process improvement and risk reduction. The Six Sigma methodology focuses on identifying and reducing variations within processes to achieve a near-perfect level of quality. By utilizing statistical tools and techniques, Six Sigma aims to achieve a level of performance where the occurrence of defects or errors is limited to less than 3.4 per million opportunities [7–9]. Although initially developed within the manufacturing industry, Six Sigma has successfully been applied in various service sectors, including healthcare, with remarkable results. In medical laboratories, Six Sigma metrics [7–9] was adopted by clinical chemistry laboratories first and later by other testing domains such as hematology and immunology [10]. By employing Six Sigma principles, these medical laboratories were able to minimize errors, reduce turnaround times, optimize resource allocation, and ultimately improve patient care outcomes.

Six Sigma can be used to decide on the best Westgard rule by judging the performance of a process against a reference method and assessing the quality of laboratory processes, thereby identifying processes needing improvement. As demonstrated by Litten [10], the implementation of a Six Sigma-designed QC programme can result in fewer controls per run, fewer false rejections, simpler Westgard rules, and a 45% saving on laboratory reagents and supplies. Infectious serology testing in Blood screening laboratories is yet to modernize its SQC practices and reap the benefits of Sigma metrics, as the others have.

The application of six sigma in blood screening laboratories presents constraint by various technical issues such as the serology screening assays being qualitative rather than quantitative. Besides that, there are methodology differences in serology tests available and lot-to-lot differences in reagent lots, which limits the ability to define performance specifications for serology testing [17]. Our study results also confirmed the impact of reagent lots changes on the assay performance. However, the lots performed within the manufacturer’s specifications and had passed lot to lot evaluation. Besides that, the observed changes in IQC were clinically unimportant. Peer group comparison and proficiency testing (PT) results between same assays and analysers could be utilized as an alternative to establish performance specifications. But not every laboratory has access to such data and PT programs might not provide s/co ratio for qualitative assays. Thus, we computed Sigma-metric with and without bias both. Bias was calculated using peer group data. However, the Sigma-metric remained >6 when calculated using bias. There are a few caveats when utilizing peer group data in this study. The EQA used is a qualitative program, and the measurable values are not evaluated. There are only three survey events per year, with a panel of ten samples per event. However, there is only one or two reactive samples per panel per analyte. The few EQA samples available is a limitation, leading to a large uncertainty in the bias estimate. Thus, it is acceptable to calculate the Sigma-metric without bias to begin with. This calculation could yield a Sigma-metric that is too high (i.e., an optimistic estimate of quality). Nonetheless, if Sigma is low (<3 when bias is excluded), it’s sufficient to indicate the test system is high risk. Both Sigma values with and without bias were world class in this study. We calculated TEa using observed and expected mean which didn’t show any significant difference for HBsAg and anti-HCV. It is evident that closer the observed mean is to expected mean, the lesser difference will be seen between observed and expected TEa. It is worth mentioning that control materials with high averages will offer higher TEa and thus falsely indicating a high sigma metric when using this method which is another study limitation. Thus, control materials with target averages closer to cut-off (1.5–3 times) should be preferred to overcome this issue.

By utilizing the insights gained from other laboratories in clinical chemistry and hematology, we endeavored to adapt and use the six-sigma model to evaluate the analytical performance of serology screening analytes in blood screening laboratories and design risk-based SQC strategies and quality improvement measures and thereby, provide more accurate and reliable analytical results for clinical application. Our results (Sigma >6) confirmed the world class performance of the analytes studied. HBsAg had one out-of-control IQC event (1:3s) resulted from wrong QC loading on Analyer 2 in April, which was removed from calculations. This random error would have been picked by Westgard Sigma rules if applied in the laboratory. Our results are in align with clinical observations of transfusion safety and as reported by other studies [18–20].

According to Westgard Sigma rules [21], QC rules could be simplified in our laboratory to 1:3s rule with N = 2 per run applied across all 3 blood screening analytes. This should result in fewer false rejections (pfr <0.01) and re-runs as shown in Fig 1. Our laboratory uses 1:2s and 10x as warning, 1:3s and 2:2s (for positive QC) as rejection rules. With these rules, there were a total of 111 (38 in February, 60 in March, and 13 in April) out-of-control events in the laboratory during the study period. On the other hand, with the implementation of Westgard Sigma rules, there would have been only 15 (2 in February, 11 in March, and 2 in April) out-of-control events. Overall, there will be a 87% reduction in out-of-control events when switching from Westgard classic rules to Westgard sigma rules. Not only, this reduces false rejections but also saves cost and time spent in re-runs and troubleshooting. It is important to highlight that this reduction can be observed only when laboratory QC limits are applied, not with Manufacturer’s limits.

It is important to note that while sigma calculations provide valuable insights, they are not standalone solutions. They should be coupled with a thorough analysis of the underlying processes, root cause identification, and the implementation of appropriate improvement strategies. Sigma calculations serve as a tool to monitor progress, set goals, and drive continuous improvement efforts within blood screening laboratories.

In conclusion, the study shows that using Six Sigma metrics to evaluate serology assays for HCV, HBV and HIV can significantly reduce errors and improve process efficiency. Simplifying QC rules to the 1:3s rule with N = 2 per run could reduce out-of-control events by 87%, thereby optimizing SQC and reducing the risk of false-negative or false-positive results in blood screening.

## Supporting information

S1 Table Month-wise N, Mean, SD, CV% and TEa% (expected) for 3 Serology analytes IQC. (XLSX)

S2 Table Month-wise number of IQC Out-of-control events for 3 Serology analytes on Analyzer 1–6. (XLSX)
